# Immunohistochemical subtypes predict the clinical outcome in high-risk node-negative breast cancer patients treated with adjuvant FEC regimen: results of a single-center retrospective study

**DOI:** 10.1186/s12885-015-1746-3

**Published:** 2015-10-14

**Authors:** S. Rahal, J M Boher, J M Extra, C. Tarpin, E. Charafe-Jauffret, E. Lambaudie, R. Sabatier, J. Thomassin-Piana, A. Tallet, M. Resbeut, G. Houvenaeghel, L. Laborde, F. Bertucci, P. Viens, A. Gonçalves

**Affiliations:** 1 Department of Medical Oncology, Institut Paoli-Calmettes, 232 Bd. Sainte-Marguerite, 13009 Marseille, France; 2 Department of Biostatistics, Institut Paoli-Calmettes, Marseille, France; 3 Department of Biopathology, Institut Paoli-Calmettes, Marseille, France; 4 Department of Surgical Oncology, Institut Paoli-Calmettes, Marseille, France; 5 Department of Radiation Oncology, Institut Paoli-Calmettes, Marseille, France; 6 Data Management and Analysis Center, Institut Paoli-Calmettes, Marseille, France; 7 Centre de Recherche en Cancérologie de Marseille, U1068 INSERM, U7258 CNRS, Marseille, France; 8 Aix-Marseille University, Marseille, France

**Keywords:** Breast cancer, Adjuvant chemotherapy, Anthracycline, Taxane, Peritumoral vascular invasion, Molecular subtypes, Prognostic factors, Node-negative

## Abstract

**Background:**

Anthracycline-based adjuvant chemotherapy improves survival in patients with high-risk node-negative breast cancer (BC). In this setting, prognostic factors predicting for treatment failure might help selecting among the different available cytotoxic combinations.

**Methods:**

Between 1998 and 2008, 757 consecutive patients with node-negative BC treated in our institution with adjuvant FEC (5FU, epirubicin, cyclophosphamide) chemotherapy were identified. Data collection included demographic, clinico-pathological characteristics and treatment information. Molecular subtypes were derived from estrogen receptor (ER), progesterone receptor (PR), human epidermal growth factor receptor 2 (HER2) status and Scarff-Bloom-Richardson (SBR) grade. Disease-free survival (DFS), distant disease-free survival (DDFS) and overall survival (OS) were estimated using the Kaplan-Meier Method, and prognostic factors were examined by multivariate Cox analysis.

**Results:**

After a median follow-up of 70 months, the 5-year DFS, DDFS and OS were 90.6 % (95 % confidence interval (CI): 88.2–93.1), 92.8 % (95 % CI: 90.7–95) and 95.1 % (95 % CI, 93.3–96.9), respectively. In the multivariate analysis including classical clinico-pathological parameters, only grade 3 maintained a significant and independent adverse prognostic impact. In an alternative multivariate model where ER, PR and grade were replaced by molecular subtypes, only luminal B/HER2-negative and triple-negative subtypes were associated with reduced DFS and DDFS.

**Conclusions:**

Node-negative BC patients receiving adjuvant FEC regimen have a favorable outcome. Luminal B/HER2-negative and triple-negative subtypes identify patients with a higher risk of treatment failure, which might warrant more aggressive systemic treatment.

**Electronic supplementary material:**

The online version of this article (doi:10.1186/s12885-015-1746-3) contains supplementary material, which is available to authorized users.

## Background

With more than 1.3 million of new cases annually and nearly 465,000 deaths, breast cancer (BC) is still the leading cause of cancer mortality among females in the world. In France, its incidence has been rising during the last 25 years and 53,000 new cases of BC were diagnosed in 2011. Adjuvant chemotherapy improves survival in early BC and is considered as one of the most significant therapeutic achievements in this disease during the last decades [1]. Initially, polychemotherapy, notably cyclophosphamide-methotrexate-5FU (CMF) regimen, was shown to significantly decrease relapses and deaths over monochemotherapy or no post-operative treatment at all [2, 3]. A few years later, anthracycline-based regimens were shown to further improve outcome over CMF, these effects being regarded as largely independent of main tumor characteristics such as estrogen receptor (ER) status, lymph node status and associated endocrine therapy [1]. Therefore, at the end of 90’s, cyclophosphamide-epirubicin-5FU (FEC) regimen became the reference combination in France for adjuvant chemotherapy in early BC, epirubicin 100 mg/m^2^ being shown as more effective than 50 mg/m^2^ in a comparative randomized trial [4]. More recently, taxanes were incorporated to adjuvant anthracycline-based chemotherapy, with demonstrated benefits in disease-free survival (DFS) and overall survival (OS) when compared to anthracycline-based combinations in various comparative randomized trials [5, 6] and meta-analysis [7].

Most of the above described results were obtained from clinical trials enrolling essentially node-positive BC patients, but several studies also demonstrated a benefit for adjuvant chemotherapy in patients with high-risk, node-negative BC [8, 9], which was clearly confirmed by meta-analyses [3, 10]. When focusing specifically on anthracyline/taxane-based combinations, most of randomized trials enrolled only node-positive BC, while only few of them also included a limited number of node-negative BC. In fact, only two randomized phase III trials evaluating concomitant or sequential anthracyline/taxane-based combinations in a pure population of node-negative BC were recently reported [11, 12], both showing a benefit in DFS, but no clear superiority in OS. Thus, regarding to the significant increase in costs and acute toxicities, the use of these anthracyline/taxane-based regimens in node-negative BC remains controversial.

Here, we have conducted a retrospective analysis of a large single-center cohort of patients with high-risk node-negative BC treated with adjuvant FEC chemotherapy. Our primary objective was to identify prognostic factors for DFS, which might help better defining node-negative BC patients candidate to the most aggressive and costly anthracycline/taxane combinations. Specifically, we have examined the prognostic impact of clinico-pathological parameters classically used to indicate adjuvant chemotherapy and that of immunohistochemically (IHC)-defined molecular subtypes of BC. Our secondary objectives were to describe OS and distant-disease free survival (DDFS) in this population. An additional secondary objective was to derive prognostic factors for DDFS.

## Methods

### Patient population

The study population was identified from our prospectively maintained institutional database. This institutional clinical data base on breast cancer is operated by DATA MANAGEMENT AND ANALYSIS CENTER (DMAC), approved for its data management skills as data processing centers by INCa (French National Institute for Cancer) in 2007. DMAC is also ISO 9001 certified. Standard operating procedures are applied to collect, check and use data in the quality management system framework. Data are entered (source is medical records) in an ORACLE database by Clinical Research Assistant.

**Fig. 1:**
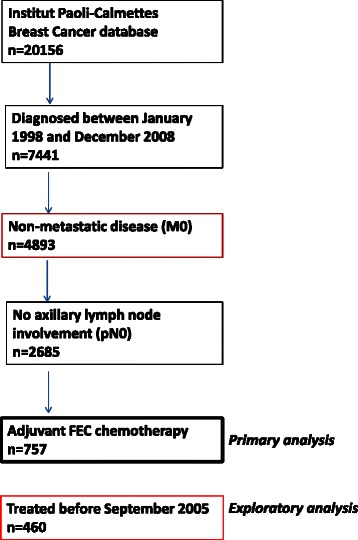
Flow diagram

The following inclusion criteria were used : all consecutive women diagnosed with invasive BC over a 11-year period between January 1998 and December 2008, treated with primary surgery (tumor resection and axillary staging by sentinel lymph node biopsy (SLNB) and/or axillary lymph node dissection (ALND)), pathologically node-negative (pN0) and treated with adjuvant FEC chemotherapy (at least 4 cycles of FEC50,75 or 100). Exclusion criteria were metastatic disease, node-positive or nodal status unknown or patient receiving neo-adjuvant treatment. A flow diagram of the population selection steps is provided in Fig. 1.

The study was approved by the Institut Paoli-Calmettes (IPC) Institutional Review Board (IRB, Comité d’Orientation Stratégique, COS). According to national guidelines, the IRB considered that there was no need for collecting informed consent for this kind of database study. Data were analyzed after all patient information have been fully anonymized.

### Clinical and biological variables

The following patient’s baseline characteristics were collected: age, type of surgery and axillary lymph node staging, pathological tumor size, histologic subtype, Scarf-Bloom-Richardson (SBR) grade (tubule formation, nuclear pleomorphism and mitotic count), peritumoral vascular invasion (PVI), IHC status for standard prognostic biomarkers : estrogen receptor (ER), progesterone receptor (PR) and human epidermal growth factor receptor 2 (HER2). For ER and PR, cases with 10 % or more positive staining were considered as positive. Hormone receptors (HoR) were considered as positive when ER and/or PR were positive. For HER2, the cases with 3+ staining by IHC and/or amplification by in situ hybridization method were considered as positive.

Five molecular subtypes were defined according to clinico-pathologic criteria. Because information on Ki-67 was only partially available, we used SBR grade to capture cell proliferation, as previously described [13]. The following definitions were used: triple-negative (HoR-negative/HER2-negative/all grades), HER2-positive (HoR-negative/HER2-positive/all grades), luminal (HoR-positive), which was divided in luminal A (HoR-positive/HER2-negative/SBR grade 1 or 2), luminal B/HER2-negative (HoR-positive/HER2-negative/SBR grade 3), luminal B/HER2-positive (HoR-positive/HER2-positive, all grades).

### Treatment procedures

Adjuvant chemotherapy was indicated for pN0 patients in case of high-risk features, defined as: pathological tumor size ≥15 mm, HoR-negative tumors, SBR grade 2 or 3, PVI, or age <40. FEC regimen was started within 12 weeks of surgery and included fluorouracil 500 mg/m^2^, epirubicin 50-75-100 mg/m^2^, cyclophosphamide 500 mg/m^2^, administered every 21 days for 4–6 cycles. Following completion of chemotherapy, patients were given adjuvant radiotherapy when appropriate and hormonal therapy in case of HoR-positivity (tamoxifen or aromatase inhibitors). From 2005, patients with HER2-positive BC were offered adjuvant trastuzumab after completion of radiotherapy.

### Statistical analyses

The primary objective of this study was to identify prognostic factors for disease-free survival (DFS). Secondary objectives included description of distant DFS (DDFS) and overall survival (OS), as well as an exploratory analysis of prognostic factors for DDFS. Due to the low number of death events, no prognostic analysis was conducted for OS. DFS was defined as the time from diagnosis to local, node, contralateral or distant relapse or death from any cause. DDFS was defined as the time from diagnosis to distant relapse or death from any cause. OS was defined as the time from diagnosis to death from any cause. For patients who remained alive and disease-free, data were censored at the date of the last follow up.

Descriptive statistics were used to describe the categorical (counts and frequency) and continuous (median and ranges) variables. DFS, DDFS and OS rates were estimated using the Kaplan–Meier method and compared with the log-rank test. Multivariate analysis was conducted using Cox’s proportional hazard regression model. All statistical tests were two-sided. The level of statistical significance was set at a P-value of 0.05. Statistical analyses were carried out with the R software version 2.15.2. We followed the “Strengthening the Reporting of Observational Studies in Epidemiology (STROBE)” guidelines [14].

## Results

### Patient’s characteristics

A total of 757 patients were identified (Fig. 1), the characteristics of which are presented in Table 1. Median age was 53 years (range, 24–79), more than half of patients had pT1 tumor (52 %), and most of tumors were HoR-positive (72 %), ductal histologic type (79 %), and 1 or 2 SBR grade (62 %). HER2 status was available for 80 % of cases and was positive in 16 % of those cases. Most of patients had breast-conserving surgery (73 %), received adjuvant radiation therapy (90 %) and hormonal therapy (70 %). Almost all patients received FEC100 regimen (94 %), FEC75 or FEC50 being usually administered if age was more than 70. Of note, 41 % of patients with HER2-positive documented BC received trastuzumab after chemotherapy and radiotherapy completion, corresponding to 85 % of HER2-positive cases diagnosed after September 2005, when trastuzumab became available in the adjuvant setting in France, but less than 5 % of those diagnosed before this date. Reasons for not receiving trastuzumab after September 2005 were: heart failure (*n* = 1), patient refusal (*n* = 1), discordant or equivocal pathological result (*n* = 2), unknown (*n* = 3).

**Table 1:** Patient characteristics

Age (Years)	Median [range]	53 (24–79)
	<35 (%)	30 (4 %)
	≥70 (%)	43 (6 %)
Type of surgery	Lumpectomy	552 (73 %)
	Complete mastectomy	205 (27 %)
Axillary management	Lymph node dissection	395 (52 %)
	Sentinel lymph node biopsy	362 (48 %)
Histological type	Invasive ductal carcinoma	599 (79 %)
	Invasive lobular carcinoma	66 (9 %)
	Other invasive carcinoma	92 (12 %)
Pathological tumor size (pT)	pT1a-b	63 (8 %)
	pT1c	329 (44 %)
	pT2	320 (43 %)
	pT3-T4	36 (5 %)
SBR grade	1	132 (18 %)
	2	331 (44 %)
	3	285 (38 %)
	NA	9
Peritumoral vascular invasion (PVI)	No	558 (76 %)
	Yes	174 (24 %)
	NA	25
Hormone receptors		
ER	Negative	229 (31 %)
	Positive	506 (69 %)
	NA	32
PR	Negative	287 (39 %)
	positive	441 (61 %)
	NA	29
ER or PR	Negative	208 (28 %)
	Positive	527 (72 %)
	NA	22
HER2 status	Positive	96 (16 %)
	Negative	508 (84 %)
	NA	153
IHC subtypes	Triple-negative	127 (21 %)
	Luminal A	290 (49 %)
	Luminal B/HER2-negative	82 (14 %)
	Luminal B/HER2-positve	58 (10 %)
	HER2	37 (6 %)
	NA	163
Adjuvant chemotherapy	FEC 100	716 (94 %)
	FEC 50/75	41 (6 %)
Adjuvant hormone therapy	Yes	532 (70 %)
	No	224 (30 %)
	NA	1
Adjuvant radiation therapy	Yes	681 (90 %)
	No	76 (10 %)
Adjuvant trastuzumab (HER2-positive)	Yes	40 (41 %)
	No	56 (59 %)

### Outcome

**Fig. 2:**
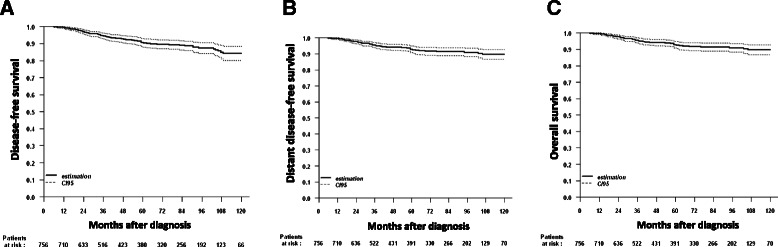
Disease-free survival (**a**), distant disease-free survival (**b**) and overall survival (**c**) in high-risk node-negative early breast cancer patients receiving adjuvant FEC

The estimated median follow-up was 70 months (95 % confidence interval (CI): 63.5–75.0). Sixty-nine DFS events, including 38 distant metastases and 39 deaths were observed. The estimated 5-year DFS, primary endpoint of this study, was 90.6 % (95 % CI: 88.2–93.1; Fig. 2A). Regarding the secondary outcome endpoints, the estimated 5-year DDFS was 92.8 % (95 % CI: 90.7–95; Fig. 2B) and 5-year OS was 95.1 % (95 % CI: 93.3–96.9; Fig. 2C).

### Clinico-pathological predictors of DFS

**Fig. 3:**
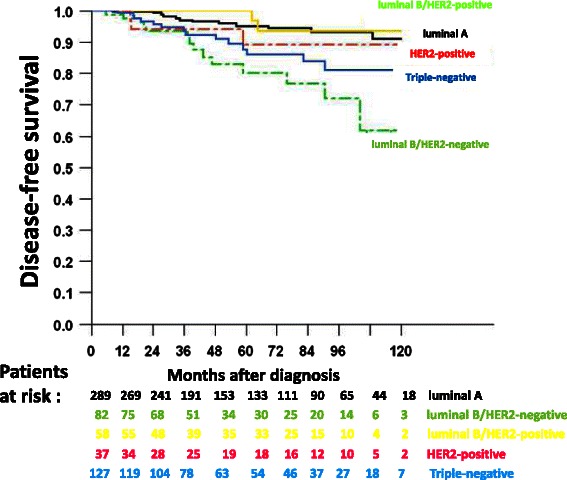
Disease-free survival according to immunohistochemical subtypes

In the univariate analysis, the following parameters were associated with a reduced DFS (Table 2): SBR grade 3 (hazard ratio (HR) =1.8, 95 % CI [1.1–2.9], p = 0.0138), PVI (HR = 2.3, 95 % CI [1.4–3.7], p = 0.0007), and IHC subtypes (p = 0.0006). As for the latter, luminal B/HER2-negative (HR = 4.3, 95 % CI [2–9.1]) and triple-negative (HR = 2.6, 95 % CI [1.2–5.5]) subtypes were associated with an adverse outcome (Fig. 3). Of note, pathological tumor size, HER2 and ER status were not prognostic in this treated population. In the multivariate analysis, only grade 3 (HR = 2.8, 95 % CI [1.4–5.4], p = 0.0027) retained an independent prognostic value (Table 3). In an alternative multivariate Cox model in which ER, PR and grade were replaced by IHC subtypes, only luminal B/HER2-negative (HR = 4.25, 95 % CI [1.9–9.15], p = 0.0002) and triple-negative (HR = 2.5, 95 % CI [1.15–5.4], p = 0.0201) subtypes were independently associated with an adverse outcome (Table 3).

**Table 2:** Prognostic factors for disease-free survival (DFS): univariate analysis

	*N*	5-Year DFS (%) (95 % CI)	HR (95 % CI)	*p*-value^a^
Age				
< 35	30	82.6 [68.2–100]	1	
≥ 35	726	90.9 [88.5–93.4]	0.5 [0.2–1.3]	0.136
SBR Grade				
1–2	462	93.2 [90.5–95.9]	1	
3	285	86 [81.4–91]	1.8 [1.1–2.9]	0.0138
Pathological tumor size				
pT1	391	91.2 [87.9–94.5]	1	
pT2	320	90 [86.2–94]	1.1 [0.7–1.8]	
pT3-T4	36	88.2 [78–99.8]	1.1 [0.4–3]	0.9514
PVI				
No	558	92.3 [89.7–95]	1	
Yes	178	85 [79–91.4]	2.3 [1.4–3.7]	0.0007
Hormone receptors				
Negative	208	86.8 [81.6–92.4]	1	
Positive	526	92.5 [89.9–95.2]	0.7 [0.4–1.1]	0.1059
HER2				
Negative	507	91 [88–94.1]	1	
Positive	96	95.7 [91–100]	0.6 [0.2–1.6]	0.376
IHC subtypes				
Luminal A	289	95.1 [92.1–98.2]	1	
Luminal B/HER2-negative	82	80.1 [69.8–92]	4.3 [2–9.1]	
Luminal B/HER2-positive	58	100 [100–100]	0.7 [0.2–3.3]	
HER2	37	89 [77.6–100]	1.8 [0.5–6.2]	
Triple-negative	127	87.6 [80.7–95]	2.6 [1.2–5.5]	0.0006

*HR* hazard ratio, *PVI* peritumor vascular invasion

^a^unadjusted log-rank test

**Table 3:** Prognostic factors for disease-free survival: multivariate analysis

	Model 1 (*N* = 570)		Model 2 (*N* = 575)	
	HR (95 % CI)	*p*-value^a^	HR (95 % CI)	*p*-value^a^
Age				
< 35	1		1	
≥ 35	0.5 [0.1–2.3]	0.581	0.5 [0.1–.396]	0.440
SBR Grade				
1–2	1		NA	
3	2.8 [1.4–5.4]	0.0027	NA	
Pathological tumor size				
pT1	1		1	
pT2	0.9 [0.5–1.7]	0.897	0.9 [0.5–1.7]	0.916
pT3-T4	0.4 [0.05–3.3]	0.431	0.4 [0.06–3.4]	0.447
PVI				
No	1		1	
Yes	1.5 [0.8–3.0]	0.175	1.3 [0.7–2.6]	0.357
Hormone receptors				
Negative	1		NA	
Positive	1.0 [0.5–2.2]	0.818	NA	
HER2				
Positive	1		NA	
Negative	0.5 [0.2–1.4]	0.239	NA	
IHC subtypes				
Luminal A	NA		1	
Luminal B/HER2-negative	NA		4.2 [1.9–9.1]	0.0002
Luminal B/HER2-positive	NA		0.7 [0.1–3.4]	0.721
HER2	NA		1.76 [0.4–6.1]	0.38
Triple-negative	NA		2.5 [1.1–5.4]	0.0201

*HR* hazard ratio, *PVI* peritumor vascular invasion

^a^Wald test

Since a large part of patients with documented HER2-positive BC received adjuvant trastuzumab after September 2005, we performed an exploratory analysis focused on patients treated before this period (*n* = 460). In this subset of patients, HER2 was still not significantly associated with DFS, and the same variables (grade 3, PVI, IHC subtypes) were found to be predictive in the univariate analysis (See Additional file 1). In the multivariate analysis, SBR grade 3 remained significantly associated with poor DFS, while a similar trend was observed for PVI (See Additional file 2). Once again, IHC subtypes (Luminal B/HER2-negative and, with borderline significance, triple-negative), instead of SBR grade, HER2 and HR, were independently associated with DFS (Additional file 2: Table S2).

### Clinico-pathological predictors of DDFS

When looking at variables associated with DDFS, SBR grade 3 (HR = 2.8, 95 % CI [1.6–4.9], p = 0.0002), PVI (HR = 2.2, 95 % CI [1.3–4], p = 0.0042), lack of hormone receptor expression (HR = 0.6, 95 % CI [0.3–1], p = 0.049), and luminal B/HER2-negative (HR = 4.6, 95 % CI [1.8–11.7]) and triple-negative (HR = 3.3, 95 % CI [1.3–8.2]) IHC subtypes (p = 0.0059) were associated with reduced DDFS in the univariate analysis (Table 4). In the multivariate analysis, only grade (HR = 3.4 95 % CI [1.5–7.6], p = 0.0027) had an independent value (Table 5). Again, when replacing hormone receptor and grade by IHC subtypes, only the latter had an independent prognostic value, with luminal B/HER2-negative and triple-negative subtypes being associated with an adverse outcome (Table 5).

**Table 4:** Prognostic factors for distant disease-free survival: univariate analysis

	N	5-Year DDFS (%) (95 % CI)	HR (95 % CI)	*p*-value^a^
Age				
< 35	30	82.6 [68.2–100]	1	
≥ 35	726	93.3 [91.2–95.4]	0.5 [0.2–1.3]	0.1223
SBR Grade				
1–2	462	96 [94–98]	1	
3	285	87.4 [82.9–92.1]	2.8 [1.6–4.9]	0.0002
Pathological tumor size				
pT1	391	93.6 [90.9–96.5]	1	
pT2	320	92.3 [88.9–95.7]	1.2 [0.7–2]	
pT3-T4	36	88.2 [78–99.8]	1.6 [0.6–4.7]	0.6395
PVI				
No	558	94.2 [92–96.5]	1	
Yes	178	88.8 [83.5–94.5]	2.2 [1.3–4]	0.0042
Hormone receptors				
Negative	208	88 [83–93.4]	1	
Positive	526	94.7 [92.5–96.9]	0.6 [0.3–1]	0.049
HER2				
Negative	507	93.2 [90.5–95.9]	1	
Positive	96	95.7 [91–100]	0.9 [0.3–2.2]	0.7719
IHC subtypes				
Luminal A	289	96.8 [94.5–99.2]	1	
Luminal B/HER2-negative	82	85.5 [76.3–95.8]	4.6 [1.8–11.7]	
Luminal B/HER2-positive	58	100 [100–100]	1.2 [0.2–5.5]	
HER2	37	89 [77.6–100]	2.9 [0.8–11.1]	
Triple-negative	127	89 [82.5–96]	3.3 [1.3–8.2]	0.0059

*HR* hazard ratio, *PVI* peritumor vascular invasion

^a^unadjusted log-rank test

**Table 5:** Prognostic factors for distant disease-free survival: multivariate analysis

	Model 1 (*N* = 570)		Model 2 (*N* = 575)	
	HR (95 % CI)	*p*-value^a^	HR (95 % CI)	*p*-value^a^
Age				
< 35	1		1	
≥ 35	0.4 [0.1–2.1]	0.344	0.4 [0.09–1.8]	0.251
SBR Grade				
1–2	1		NA	
3	3.4 [1.5–7.6]	0.0027	NA	
Pathological tumor size				
pT1	1		1	
pT2	1.0 [0.5–2.0]	0.932	1.0 [0.5–2.1]	0.847
pT3-T4	0.7 [0.09–5.3]	0.733	0.7 [0.09–5.5]	0.763
PVI				
No	1		1	
Yes	1.7 [0.8–3.6]	0.155	1.5 [0.7–3.2]	0.275
Hormone receptors				
Negative	1		NA	
Positive	0.9 [0.4–2.0]	0.865	NA	
HER2				
Positive	1		NA	
Negative	0.7 [0.2–2.0]	0.605	NA	
IHC subtypes				
Luminal A	NA		1	
Luminal B-HER2–negative	NA		4.4 [1.7–11.5]	0.0018
Luminal B-HER2-positive	NA		1.2 [0.2–5.9]	0.775
HER2	NA		2.8 [0.7–10.9]	0.118
Triple-negative	NA		3.4 [1.3–8.7]	0.0074

*HR* hazard ratio, *PVI* peritumor vascular invasion

^a^Wald test

## Discussion

In this paper, we have examined the clinical outcome of a large, single-centre, retrospective cohort of patients with high-risk node-negative early BC receiving post-operative FEC-based chemotherapy. With a 5-year DFS of more than 90 %, and a 5-year OS of more than 95 %, overall prognosis was considered to be excellent, but some subsets of patients were identified as displaying a less favorable outcome. Namely, the presence of a SBR grade 3 still predicted for a higher risk of relapse. A similar independent dismal prognosis was identified for luminal B/HER2-negative and triple-negative subtypes, suggesting that these patients might be the best candidate to more aggressive adjuvant chemotherapy, including the more aggressive and costly anthracycline and taxane-based combination.

Histologic grade is a semi-quantitative estimation of the degree of tubular or gland formation, nuclear polymorphism and mitotic count [15], the independent prognostic impact of which was recurrently demonstrated in various settings [16], including node-negative or node-positive disease [17]. Accordingly, high grade has been considered as a major determinant for adjuvant chemotherapy decision, which is related at least partially to its dependency on cell proliferation and mitosis. Here, we have shown that SBR grade still retains an independent adverse prognostic value, in the context of node-negative BC patients receiving anthracycline-based adjuvant chemotherapy. Thus, even though it does not seem to predict a specific sensitivity to a particular class of drug, it may indicate a subset of BC patients in which adding taxanes to anthracylines may be of interest in spite of the absence of lymph node invasion.

PVI was shown to be an adverse prognostic parameter in early BC, notably in node-negative BC, where it has been considered as a surrogate for occult vascular dissemination [18]. Its prognostic value has been also described within the different breast cancer subtypes [19, 20]. In our study, whereas it was consistently associated with DFS and DDFS, it was not retained as an independent prognosticator in all multivariate models tested. That suggests that, in the context anthracycline-based adjuvant CT, the unfavorable prognostic impact of PVI is not as important as proliferation status and subtypes. This lack of prognostic value in multivariate analysis including breast cancer subtypes could also be explained by the fact that PVI was recently shown to correlate with the luminal B/HER2-negative subtype [21].

Of note, age and pathological tumor size were not associated with survival, although being classical prognosticators currently used to recommend adjuvant chemotherapy. Thus, following anthracycline-based adjuvant chemotherapy, prognosis and therefore the potential need for taxane-based treatment, may more result from biological rather than from pure clinical features.

Notably, the lack of hormone receptors and HER2 overexpression were not individually associated with a significantly higher risk of relapse. This may be related to the classically documented efficacy of anthracycline-based adjuvant chemotherapy in HR-negative tumors [1, 3]. An explanation for the relative favorable outcome of patients with HER2 overexpression may be that a significant fraction of those patients received, after completion of chemotherapy and radiotherapy, adjuvant trastuzumab, the impact of which was largely demonstrated during the last 10 years [22, 23]. However, a sensitivity analysis focused on patients treated before 2005 September (date of initiation of adjuvant trastuzumab in HER2-positive BC at our institution) did not show a significant adverse impact of HER2-overexpression in this population. An alternative hypothesis could be the suspected link between *HER2* amplification and sensitivity to anthracycline-based regimen [24, 25], possibly due to frequent co-amplification or deletion of *TOP2A*, the gene encoding topoisomerase IIα protein, which is a supposed intracellular target for anthracyclines [26].

Results from transcriptomic studies have shown that BC is an heterogeneous disease that can be divided in at least 5 molecular subtypes (basal-like, HER2-enriched, luminal A, luminal B and normal-like) with specific gene expression patterns, distinct clinical outcome and sensitivity to systemic treatments [27]. Accordingly, it has been hypothesized that identifying these BC subtypes in the clinic could help better tailoring individual therapeutic choices. Since molecular subtypes are only precisely identified by sophisticated gene expression profiling technologies not readily applicable to clinical samples in routine, it has become increasingly popular to approximate these subtypes using IHC evaluation of ER, PR and HER2 expression, combined to a surrogate of the proliferation status such as Ki-67 expression or grade. The main objective of this subtype-based classification has been to identify patients with low or no tumor burden in the axillary lymph node and a relatively indolent disease, who could be spared from adjuvant cytotoxic treatment [28]. Thus, according to this classification, most of luminal A tumors need endocrine but not cytotoxic treatment, whereas adjuvant chemotherapy is recommended in tumors identified as triple-negative, luminal B (followed by endocrine treatment), and HER2 (with trastuzumab). In this study, we have used IHC subtypes to identify patients with node-negative BC receiving adjuvant FEC who may have still a significant residual risk of relapse. Triple-negative and luminal-B/HER2-negative subtypes displayed the worst outcome, including a distant relapse risk which was higher than 10 % in both cases, theoretically justifying a more aggressive systemic treatment. Triple-negative status is a very well-known poor prognostic factor in early BC and some retrospective analyses of randomized studies have shown that this subset of tumors may derive a pronounced benefit from taxane addition, including node-negative BC [29–31]. However, the triple-negative subtype has been shown to be heterogeneous at the molecular level, including various subtypes with distinct clinical behavior and phenotype of drug sensitivity [32]. Specifically, recent data have identified a favorable prognosis in triple-negative BC receiving anthracyline-based adjuvant chemotherapy and displaying a high level of lymphocytic infiltration [33, 34].

Notably and surprisingly, the luminal B/HER2-negative subtype was found to be even more aggressive, with a 5-year risk of relapse of 20 %, including a risk of distant relapse of nearly 15 %. The aggressive clinical behavior of Luminal B tumors is well known and their prognosis has been considered as similar to that of HER2-enriched and basal-like groups [35]. However, while triple-negative and Luminal B/HER2-positive tumors might derive higher benefits from anthracyclines and/or anti-HER2 adjuvant treatment, some Luminal B/HER2-negative could be sub-optimally treated with anthracycline-only regimen. Thus, these results suggest that these tumors should also be candidate to more aggressive chemotherapy with addition of taxanes to anthracycline-based combination. Of note, in the French PACS 01 study, which compared in node-positive BC treated in the adjuvant setting 6 cycles of FEC100 versus 3 cycles FEC100 followed by 3 cycles of docetaxel, luminal B tumors were shown to derive the highest benefit from taxane addition [36].

Even though they were not found to display a significantly higher risk of relapse in our study (which could be due to the relatively limited sample size of this subgroup), HER2-positive subtypes are also pragmatic candidates to taxane, since it is a way to earlier initiate trastuzumab, either after a limited anthracycline-based sequence or at the beginning of chemotherapy in the context of anthracycline-free combinations. Indeed, early initiation of trastuzumab has been associated with better outcome, while reducing anthracycline exposure may limit cardiac toxicity [37]. Finally, with a risk of distant relapse of less than 4 %, luminal A BC had an excellent outcome and the most relevant question in this subset of patients, sensitive to hormone therapy, is whether these patients do actually need any cytotoxic treatment. Various multigenic signatures are now available that are specifically addressing this issue [38, 39] and the prospective validation of some of them is currently under analysis [40, 41].

This study has several strengths lying in the number of samples analyzed (more than 750), the duration of the follow-up (nearly 8 years in median) and the high-quality of data collected by a certified Data Management and Analysis Center. Weaknesses include its retrospective monocentric, non-randomized and non-comparative nature, the lack of central and ad hoc review of biological variables, the relatively high number of missing data for analysis of molecular subtypes (*n* = 163), notably the number of missing HER2 status, the discussable approximation of luminal A/B distinction based on grade only, rather than on Ki67 and the low number of death events, precluding the identification of prognostic factors for OS. Another potential bias may be the definition of high-risk features used to indicate adjuvant chemotherapy during this period (pathological tumor size ≥15 mm, HoR-negative tumors, SBR grade 2 or 3, PVI, or age <40), which is not the same as currently used. Finally, the relatively low number of HER2-positive patients receiving trastuzumab (49 %) may also affect the clinical relevance of our results in routine practice.

In spite of the above-mentioned limitations, our main result suggests that triple-negative and luminal-B/HER2-negative subtypes have a significant residual risk of relapse following FEC adjuvant chemotherapy. This may support the use of taxanes in these subsets of patients, even when node-negative, but additional researches are warranted in order to better predict a specific therapeutic benefit of this class of drugs according to the tumor features.

## Conclusions

In conclusion, our results suggest a relative favorable outcome in node-negative BC treated with FEC-based adjuvant chemotherapy. However, SBR grade 3 or triple-negative and luminal-B/HER2-negative subtypes may indicate which patients are candidates to anthracyline and taxane-based combinations.

## Additional files

Additional file 1: **Prognostic factors for disease-free survival in patients treated before 2005 September: univariate analysis.** (DOCX 15 kb)
Additional file 2: **Prognostic factors for disease-free survival in patients treated before 2005 September: multivariate analysis.** (DOCX 15 kb)

## Abbreviations

ALND: Axillary lymph node dissection
BC: Breast cancer
CI: Confidence interval
CMF: Cyclophosphamide, methotrexate, 5FU
COS: Comité d’Orientation Stratégique
DDFS: Distant disease-free survival
DFS: Disease-free survival
DMAC: Data management and analysis center
ER: Estrogen receptor
FEC: 5FU, Epirubicin, Cyclophosphamide
HER2: Human epidermal growth receptor 2
HoR: Hormone receptors
IHC: Immunohistochemistry
INCa: French National Institute for Cancer
IPC: Institut Paoli-Calmettes
IRB: Institutional Review Board
OS: Overall survival
pN0: Pathologically node-negative
PR: Progesterone receptor
PVI: Peritumoral vascular invasion
SBR: Scarff-Bloom-Richardson
SLNB: Sentinel lymph node biopsy
STROBE: Strengthening the Reporting of Observational Studies in Epidemiology
TOP2A: Topoisomerase IIα

## References

[1] Early Breast Cancer Trialists’ Collaborative Group (EBCTCG) (2005). Effects of chemotherapy and hormonal therapy for early breast cancer on recurrence and 15-year survival: an overview of the randomised trials. Lancet.

[2] Bonadonna G, Valagussa P, Moliterni A, Zambetti M, Brambilla C (1995). Adjuvant cyclophosphamide, methotrexate, and fluorouracil in node-positive breast cancer: the results of 20 years of follow-up. N Engl J Med.

[3] Early Breast Cancer Trialists’ Collaborative Group (EBCTCG) (1998). Polychemotherapy for early breast cancer: an overview of the randomised trials. Early Breast Cancer Trialists’ Collaborative Group. Lancet.

[4] French Adjuvant Study Group (2001). Benefit of a high-dose epirubicin regimen in adjuvant chemotherapy for node-positive breast cancer patients with poor prognostic factors: 5-year follow-up results of French Adjuvant Study Group 05 randomized trial. J Clin Oncol Off J Am Soc Clin Oncol.

[5] Henderson IC, Berry DA, Demetri GD, Cirrincione CT, Goldstein LJ, Martino S (2003). Improved outcomes from adding sequential Paclitaxel but not from escalating Doxorubicin dose in an adjuvant chemotherapy regimen for patients with node-positive primary breast cancer. J Clin Oncol Off J Am Soc Clin Oncol.

[6] Roché H, Fumoleau P, Spielmann M, Canon J-L, Delozier T, Serin D (2006). Sequential adjuvant epirubicin-based and docetaxel chemotherapy for node-positive breast cancer patients: the FNCLCC PACS 01 Trial. J Clin Oncol Off J Am Soc Clin Oncol.

[7] De Laurentiis M, Cancello G, D’Agostino D, Giuliano M, Giordano A, Montagna E (2008). Taxane-based combinations as adjuvant chemotherapy of early breast cancer: a meta-analysis of randomized trials. J Clin Oncol Off J Am Soc Clin Oncol.

[8] Mansour EG, Gray R, Shatila AH, Tormey DC, Cooper MR, Osborne CK (1998). Survival advantage of adjuvant chemotherapy in high-risk node-negative breast cancer: ten-year analysis--an intergroup study. J Clin Oncol Off J Am Soc Clin Oncol.

[9] Fisher B, Anderson S, Tan-Chiu E, Wolmark N, Wickerham DL, Fisher ER (2001). Tamoxifen and chemotherapy for axillary node-negative, estrogen receptor-negative breast cancer: findings from National Surgical Adjuvant Breast and Bowel Project B-23. J Clin Oncol Off J Am Soc Clin Oncol.

[10] Early Breast Cancer Trialists’ Collaborative Group (EBCTCG) (2012). Comparisons between different polychemotherapy regimens for early breast cancer: meta-analyses of long-term outcome among 100,000 women in 123 randomised trials. Lancet.

[11] Martín M, Seguí MA, Antón A, Ruiz A, Ramos M, Adrover E (2010). Adjuvant docetaxel for high-risk, node-negative breast cancer. N Engl J Med.

[12] Martín M, Ruiz A, Ruiz Borrego M, Barnadas A, González S, Calvo L (2013). Fluorouracil, doxorubicin, and cyclophosphamide (FAC) versus FAC followed by weekly paclitaxel as adjuvant therapy for high-risk, node-negative breast cancer: results from the GEICAM/2003-02 study. J Clin Oncol Off J Am Soc Clin Oncol.

[13] Von Minckwitz G, Untch M, Blohmer J-U, Costa SD, Eidtmann H, Fasching PA (2012). Definition and impact of pathologic complete response on prognosis after neoadjuvant chemotherapy in various intrinsic breast cancer subtypes. J Clin Oncol Off J Am Soc Clin Oncol.

[14] Von Elm E, Altman DG, Egger M, Pocock SJ, Gøtzsche PC, Vandenbroucke JP (2007). The Strengthening the Reporting of Observational Studies in Epidemiology (STROBE) statement: guidelines for reporting observational studies. PLoS Med.

[15] Elston CW, Ellis IO (1991). Pathological prognostic factors in breast cancer. I. The value of histological grade in breast cancer: experience from a large study with long-term follow-up. Histopathology.

[16] Rakha EA, Reis-Filho JS, Baehner F, Dabbs DJ, Decker T, Eusebi V (2010). Breast cancer prognostic classification in the molecular era: the role of histological grade. Breast Cancer Res.

[17] Mirza AN, Mirza NQ, Vlastos G, Singletary SE (2002). Prognostic factors in node-negative breast cancer: a review of studies with sample size more than 200 and follow-up more than 5 years. Ann Surg.

[18] Lee AHS, Pinder SE, Macmillan RD, Mitchell M, Ellis IO, Elston CW (2006). Prognostic value of lymphovascular invasion in women with lymph node negative invasive breast carcinoma. Eur J Cancer.

[19] Rakha EA, Martin S, Lee AHS, Morgan D, Pharoah PDP, Hodi Z (2012). The prognostic significance of lymphovascular invasion in invasive breast carcinoma. Cancer.

[20] Sabatier R, Jacquemier J, Bertucci F, Esterni B, Finetti P, Azario F (2011). Peritumoural vascular invasion: A major determinant of triple-negative breast cancer outcome. Eur J Cancer.

[21] Munzone E, Bagnardi V, Rotmensz N, Sporchia A, Mazza M, Pruneri G (2014). Prognostic relevance of peritumoral vascular invasion in immunohistochemically defined subtypes of node-positive breast cancer. Breast Cancer Res Treat.

[22] Romond EH, Perez EA, Bryant J, Suman VJ, Geyer CE, Davidson NE (2005). Trastuzumab plus adjuvant chemotherapy for operable HER2-positive breast cancer. N Engl J Med.

[23] Piccart-Gebhart MJ, Procter M, Leyland-Jones B, Goldhirsch A, Untch M, Smith I (2005). Trastuzumab after adjuvant chemotherapy in HER2-positive breast cancer. N Engl J Med.

[24] Pritchard KI, Shepherd LE, O’Malley FP, Andrulis IL, Tu D, Bramwell VH (2006). HER2 and responsiveness of breast cancer to adjuvant chemotherapy. N Engl J Med.

[25] Gennari A, Sormani MP, Pronzato P, Puntoni M, Colozza M, Pfeffer U (2008). HER2 status and efficacy of adjuvant anthracyclines in early breast cancer: a pooled analysis of randomized trials. J Natl Cancer Inst.

[26] Di Leo A, Desmedt C, Bartlett JMS, Piette F, Ejlertsen B, Pritchard KI (2011). HER2 and TOP2A as predictive markers for anthracycline-containing chemotherapy regimens as adjuvant treatment of breast cancer: a meta-analysis of individual patient data. Lancet Oncol.

[27] Perou CM, Sørlie T, Eisen MB, van de Rijn M, Jeffrey SS, Rees CA (2000). Molecular portraits of human breast tumours. Nature.

[28] Goldhirsch A, Winer EP, Coates AS, Gelber RD, Piccart-Gebhart M, Thürlimann B (2013). Personalizing the treatment of women with early breast cancer: highlights of the St Gallen International Expert Consensus on the Primary Therapy of Early Breast Cancer 2013. Ann Oncol Off J Eur Soc Med Oncol ESMO.

[29] Hayes DF, Thor AD, Dressler LG, Weaver D, Edgerton S, Cowan D (2007). HER2 and response to paclitaxel in node-positive breast cancer. N Engl J Med.

[30] Hugh J, Hanson J, Cheang MCU, Nielsen TO, Perou CM, Dumontet C (2009). Breast cancer subtypes and response to docetaxel in node-positive breast cancer: use of an immunohistochemical definition in the BCIRG 001 trial. J Clin Oncol Off J Am Soc Clin Oncol.

[31] Martín M, Rodríguez-Lescure A, Ruiz A, Alba E, Calvo L, Ruiz-Borrego M (2010). Molecular predictors of efficacy of adjuvant weekly paclitaxel in early breast cancer. Breast Cancer Res Treat.

[32] Lehmann BD, Bauer JA, Chen X, Sanders ME, Chakravarthy AB, Shyr Y (2011). Identification of human triple-negative breast cancer subtypes and preclinical models for selection of targeted therapies. J Clin Invest.

[33] Adams S, Gray RJ, Demaria S, Goldstein L, Perez EA, Shulman LN (2014). Prognostic value of tumor-infiltrating lymphocytes in triple-negative breast cancers from two phase III randomized adjuvant breast cancer trials: ECOG 2197 and ECOG 1199. J Clin Oncol.

[34] Loi S, Michiels S, Salgado R, Sirtaine N, Jose V, Fumagalli D (2014). Tumor infiltrating lymphocytes are prognostic in triple negative breast cancer and predictive for trastuzumab benefit in early breast cancer: results from the FinHER trial. Ann Oncol Off J Eur Soc Med Oncol ESMO.

[35] Ades F, Zardavas D, Bozovic-Spasojevic I, Pugliano L, Fumagalli D, de Azambuja E (2014). Luminal B breast cancer: molecular characterization, clinical management, and future perspectives. J Clin Oncol Off J Am Soc Clin Oncol.

[36] Jacquemier J, Boher J-M, Roche H, Esterni B, Serin D, Kerbrat P (2011). Protein expression, survival and docetaxel benefit in node-positive breast cancer treated with adjuvant chemotherapy in the FNCLCC-PACS 01 randomized trial. Breast Cancer Res.

[37] Perez EA, Suman VJ, Davidson NE, Gralow JR, Kaufman PA, Visscher DW (2011). Sequential versus concurrent trastuzumab in adjuvant chemotherapy for breast cancer. J Clin Oncol Off J Am Soc Clin Oncol.

[38] Van de Vijver MJ, He YD, van’t Veer LJ, Dai H, Hart AAM, Voskuil DW (2002). A gene-expression signature as a predictor of survival in breast cancer. N Engl J Med.

[39] Paik S, Shak S, Tang G, Kim C, Baker J, Cronin M (2004). A multigene assay to predict recurrence of tamoxifen-treated, node-negative breast cancer. N Engl J Med.

[40] Cardoso F, Van’t Veer L, Rutgers E, Loi S, Mook S, Piccart-Gebhart MJ (2008). Clinical application of the 70-gene profile: the MINDACT trial. J Clin Oncol Off J Am Soc Clin Oncol.

[41] Sparano JA, Paik S (2008). Development of the 21-gene assay and its application in clinical practice and clinical trials. J Clin Oncol Off J Am Soc Clin Oncol.

