# Supplementary material for: Immunohistochemical subtypes predict the clinical outcome in high-risk node-negative breast cancer patients treated with adjuvant FEC regimen: results of a single-center retrospective study
Source: BMC Cancer. 2015 Oct 14;15:697. doi: 10.1186/s12885-015-1746-3 (PMC4607139; doi:10.1186/s12885-015-1746-3)
Supplement: Additional file 1: — Prognostic factors for disease-free survival in patients treated before 2005 September: univariate analysis. (DOCX 15 kb) [file 12885_2015_1746_MOESM1_ESM.docx]

**Additional file 1** **– Prognostic factors for disease-free survival in patients treated before 2005 September: univariate analysis**

|  | N | 5-Year DFS (%)  (95%CI) | HR (95%CI) | p-value* |
| --- | --- | --- | --- | --- |
| Age  <35  > 35  SBR Grade  1-2  3  Pathological tumor size  pT1  pT2  pT3-T4  PVI  No  Yes  Hormone Receptors  No  Yes  HER2  No  Yes  IHC subtypes  Luminal A  Luminal B/HER2-negative  Luminal B/HER2-positive  HER2  Triple-negative | 18  442  287  166  236  196  27  332  110  135  309  266  51  153  38  30  21  71 | 82.4 [66.1-100]  89.7 [86.8-92.7]  92.7 [89.6-95.8]  83.3 [77.6-89.4]  89.5 [85.5-93.7]  89.8 [85.5-94.3]  84.9 [72.3-99.7]  92 [89-95]  80.9 [73.7-88.8]  86.3 [80.5-92.6]  91.2 [88-94.5]  89.6 [85.9-93.5]  96 [90.7-100]  94.5 [90.8-98.3]  73.2 [60.3-88.9]  100 [100-100]  90 [77.8-100]  87.7 [80-96.1] | 1  0.6 [0.2-1.6]  1  1.9 [1.2-3.2]  1  1 [0.6-1.6]  1.1 [0.4-3]  1  2.7 [1.6-4.5]  1  0.7 [0.4-1.3]  1  0.6 [0.2-1.6]  1  5.3 [2.4-11.9]  0.9 [0.2-4.1]  1.4 [0.3-6.5]  2.4 [1-5.5] | 0.2784  0.0009  0.9759  <0.0001  0.1059  0.3006  0.0002 |

HR=hazard ratio, PVI=peritumor vascular invasion

* unadjusted log-rank test
