# Supplementary material for: Immunohistochemical subtypes predict the clinical outcome in high-risk node-negative breast cancer patients treated with adjuvant FEC regimen: results of a single-center retrospective study
Source: BMC Cancer. 2015 Oct 14;15:697. doi: 10.1186/s12885-015-1746-3 (PMC4607139; doi:10.1186/s12885-015-1746-3)
Supplement: Additional file 2: — Prognostic factors for disease-free survival in patients treated before 2005 September: multivariate analysis. (DOCX 15 kb) [file 12885_2015_1746_MOESM2_ESM.docx]

**Additonal file 2** **– Prognostic factors for disease-free survival in patients treated before 2005 September: multivariate analysis**

|  | Model 1 (N=303) |  | Model 2 (N=306) |  |
| --- | --- | --- | --- | --- |
|  | HR (95%CI) | p-value* | HR (95%CI) | p-value* |
| Age  <35  > 35  SBR Grade  1-2  3  Pathological tumor size  pT1  pT2  pT3-T4  PVI  No  Yes  Hormone Receptors  No  Yes  HER2  Yes  No  IHC subtypes  Luminal A  Luminal B/HER2-negative  Luminal B/HER2-positive  HER2  Triple-negative | 1  0.9 [0.13-7.2]  1  3.5 [1.7-7.3]  1  0.7[0.3-1.4]  0.4[0.06-3.4]  1  1.9 [0.9-3.9]  1  1.5 [0.674-3.354]  1  0.5 [0.2-1.6]  NA  NA NA NA NA | 0.98  0.0005  0.357  0.443  0.0512  0.32  0.321 | 1  0.8[0.1-6.2]  NA  NA  1  0.70[0.3-1.4]  0.4[0.06-3.5]  1  1.6 [0.8-3.4]  NA  NA  NA  NA  1  5.1[2.2-11.7]  0.9[0.2-4.1]  1.3[0.3-6.2]  2.3[0.9-5.5] | 0.868  0.372  0.455  0.14  <0.0001  0.907  0.675  0.0529 |

HR=hazard ratio, PVI=peritumor vascular invasion

*Wald test
